# Nutrient-Derived Modulation of the Gremlin-1/BMP-4 Axis by White Tea Preserves Insulin Sensitivity During Early Diet-Induced Metabolic Dysregulation

**DOI:** 10.3390/ijms27052512

**Published:** 2026-03-09

**Authors:** Medeni Arpa, Bayram Şen, Mehtap Atak, Hülya Kılıç

**Affiliations:** 1 Clinical Biochemistry Department, Faculty of Medicine, Recep Tayyip Erdoğan University, 53020 Rize, Türkiye; medeni.arpa@erdogan.edu.tr (M.A.);; 2 Medical Biochemistry Core Laboratory, Research and Training Hospital, Recep Tayyip Erdoğan University, 53100 Rize, Türkiye

**Keywords:** BMP4, *Camellia sinensis*, Gremlin-1, GREM1, metabolic dysregulation, white tea

## Abstract

Given the increasing burden of diet-induced metabolic dysregulation, preventive nutritional strategies targeting early insulin resistance are of growing interest. The aim of this study was to evaluate the effects of white tea supplementation on body weight gain, insulin resistance, and the Gremlin-1/Bone Morphogenetic Protein-4 (BMP-4) axis in visceral adipose tissue under high-fat diet conditions in a non-obese experimental model. Thirty-two male Sprague–Dawley rats were randomized into four groups (*n* = 8/group): standard diet (control), only high-fat diet (HFD), high-fat diet plus orlistat (ORL: 30 mg/kg/day), and high-fat diet plus white tea (WT: 5 mg/kg/day). Interventions were administered once daily by oral gavage for 12 weeks. Body weight was recorded weekly. At the end of the study, serum insulin, Gremlin-1, and BMP-4 and retroperitoneal adipose tissue Gremlin-1 and BMP-4 levels were measured by ELISA. Adipose tissue GREM1 gene expression was quantified by qRT-PCR. Insulin resistance was estimated using the HOMA-IR index. Appropriate statistical analyses were conducted in line with the study design and data distribution. High-fat feeding resulted in the highest HOMA-IR values, whereas white tea supplementation reduced HOMA-IR compared to the HFD group (*p* = 0.008). Body weight gain was increased in both the HFD and ORL groups compared to the control (*p* = 0.009 and *p* = 0.012, respectively). The lowest weight gain was observed in the WT group, which was lower than the HFD group (*p* = 0.044). GREM1 expression showed a 1.92-fold increase in the HFD group relative to the control, with smaller increases in the WT and ORL groups; however, intergroup differences did not reach statistical significance (*p* = 0.063). Serum BMP-4 levels were lower in the WT group compared to the control (*p* = 0.012), while tissue BMP-4 and Gremlin-1 levels did not differ between groups. Correlation analyses revealed a moderate inverse association between serum Gremlin-1 and serum BMP-4 (rho = −0.493, *p* = 0.011) and a moderate positive correlation between serum BMP-4 and HOMA-IR (rho = 0.564, *p* = 0.003). White tea supplementation attenuated body weight gain and preserved insulin sensitivity in a non-obese high-fat diet model, as evidenced by reduced weight gain and lower HOMA-IR values compared with high-fat feeding alone. These metabolic improvements were accompanied by coordinated changes in circulating components of the Gremlin-1/BMP-4 axis, including reduced serum BMP-4 levels and associations between BMP-4, Gremlin-1, and insulin resistance. Although tissue-level alterations were modest, the observed systemic patterns are consistent with an exploratory association between white tea intake and early metabolic signaling changes; however, definitive pathway modulation cannot be inferred from the present dataset. Collectively, these findings support white tea as a nutrient-derived bioactive with preventive metabolic potential during the early stages of diet-induced metabolic dysregulation, prior to the development of overt obesity.

## 1. Introduction

High-fat dietary patterns have been extensively linked to metabolic dysregulation, which serves as a pivotal foundation for developing insulin resistance, obesity, and various cardiometabolic conditions [1]. This relationship is orchestrated through various mechanisms involving inflammatory pathways, lipid accumulation, hormonal changes, and genetic factors, which collectively highlight the detrimental impact of high-fat diets on metabolic health [2]. Even before the onset of overt obesity, alterations in glucose and lipid metabolism can emerge, characterized by impaired insulin sensitivity and progressive disruption of energy homeostasis [3]. Insulin resistance is considered a central pathophysiological feature in this early metabolic imbalance and plays a pivotal role in the transition from metabolic health to chronic metabolic disease [4]. Insulin resistance impairs the insulin-mediated regulation of glucose and lipid metabolism and causes more fat breakdown in fat tissue. This results in higher levels of free fatty acids in the blood. Excessive lipid accumulation in peripheral tissues results in metabolic inflexibility, wherein tissues exhibit a compromised ability to appropriately transition between lipid and glucose utilization [5,6].

Adipose tissue, particularly visceral adipose tissue, is not merely a passive lipid storage site but an active endocrine organ that integrates nutrient signals and modulates metabolic and inflammatory pathways through complex gene expression networks [3]. Metabolic challenges resulting from dietary intake can modify gene expression within adipose tissue, impacting adipogenesis, insulin signaling, and the remodeling of the extracellular matrix [7]. Among these, the Gremlin-1/Bone Morphogenetic Protein-4 (BMP-4) signaling axis has emerged as a relevant regulatory pathway. Gremlin-1 is a released glycoprotein that functions primarily as an antagonist of bone morphogenetic proteins, including BMP-4, by directly binding to BMP ligands and inhibiting downstream signaling. While Gremlin-1 was initially characterized for its role in embryonic development, increasing evidence indicates that it is also involved in metabolic regulation, adipose tissue remodeling, inflammation, and insulin resistance. In adipose tissue, elevated Gremlin-1 expression has been related to impaired adipocyte differentiation, altered extracellular matrix dynamics, and reduced insulin sensitivity, particularly under conditions of metabolic stress [8].

BMP-4 plays a multifaceted role in adipose tissue biology, influencing adipocyte lineage commitment, lipid metabolism, and systemic insulin sensitivity. Dysregulated BMP-4 signaling has been reported in obesity and metabolic syndrome, and circulating BMP-4 has been related to insulin resistance and adverse metabolic profiles. Given the antagonistic relation between Gremlin-1 and BMP-4, the balance of this signaling axis is recognized as a potential molecular indicator of adipose tissue dysfunction and early metabolic maladaptation [9].

Alterations in the Gremlin-1/BMP-4 axis may occur prior to the development of overt obesity, reflecting early adaptive or maladaptive responses to excess dietary fat. However, despite growing interest in this pathway, limited data are available regarding its modulation by nutrient-derived bioactive compounds, particularly during the early stages of diet-induced metabolic dysregulation. This gap highlights the relevance of investigating the Gremlin-1/BMP-4 axis as a target for preventive nutritional interventions. BMP-4 plays a role in adipocyte differentiation, insulin sensitivity, and metabolic regulation [8,9]. Dysregulation of this axis has been associated with impaired adipose tissue function and insulin resistance, suggesting that Gremlin-1 and BMP-4 may reflect metabolic responses to dietary challenges [10]. Gremlin-1 is a pleiotropic regulator that modulates fundamental biological processes across embryonic development, inflammation, angiogenesis, cancer progression, and tissue fibrosis through its context-dependent interactions with BMPs and multiple receptor-mediated signaling pathways [11,12,13,14]. Its functions are highly tissue- and state-specific, exhibiting both protective and pathogenic roles depending on factors such as oligomeric state, redox conditions, and microenvironmental cues, thereby influencing processes including immune cell recruitment, endothelial activation, tumor growth, and mesenchymal transdifferentiation [15,16,17].

White tea, derived from *Camellia sinensis*, is a minimally processed tea rich in polyphenols and other bioactive compounds [18]. Unlike traditional pharmacological interventions, white tea represents a dietary source of nutrient bioactives that may influence metabolic regulation [19]. Experimental studies on tea-derived polyphenols have demonstrated beneficial effects on insulin sensitivity, lipid metabolism, and oxidative balance, suggesting their potential to modulate metabolic signaling pathways at both systemic and tissue levels [20,21,22,23]. However, the molecular mechanisms of the preventive metabolic impact of white tea, particularly in relation to adipose tissue gene expression and signaling pathways, remain insufficiently characterized.

Current strategies aimed at preventing metabolic disorders emphasize lifestyle modification, with diet representing a cornerstone of early intervention [24]. Beyond macronutrient composition, increasing attention has been directed toward the role of nutrient-derived bioactive compounds capable of modulating metabolic pathways and gene expression. Such compounds may exert beneficial effects at early stages of metabolic dysregulation, potentially delaying or preventing the progression toward obesity and insulin resistance [25,26,27,28]. Moreover, while several studies have focused on therapeutic interventions in established obesity, fewer investigations have addressed the impact of nutrient-derived bioactives during early diet-induced metabolic alterations [29,30,31]. Understanding how such compounds influence metabolic outcomes and gene expression-related signaling pathways before the development of overt obesity is crucial for advancing preventive nutritional strategies.

Therefore, the aim of the present study was to assess the effects of white tea administration on metabolic outcomes and gene expression-related signaling under high-fat diet conditions in a non-obese experimental model. Specifically, we investigated changes in body weight, insulin resistance, and the Gremlin-1/BMP-4 axis in visceral adipose tissue, in order to elucidate whether a nutrient-derived bioactive may modulate early metabolic dysregulation at both metabolic and molecular levels.

## 2. Results

### 2.1. Experimental Groups and General Observations

All subjects successfully adhered to the established experimental procedure, and no untoward incidents were reported during the duration of the investigation. Standardized housing was provided for the rats, and all experimental interventions were administered without adverse effects. Body weight measurements and biological sample collection were completed as planned in all experimental groups. In addition, cumulative food intake differed among groups during the experimental period. Rats in the WT group exhibited approximately 30% lower total food consumption compared with the HFD group, while no overt signs of distress or feeding intolerance were observed.

### 2.2. Insulin Resistance and Body Weight Gain

**Figure 1:**
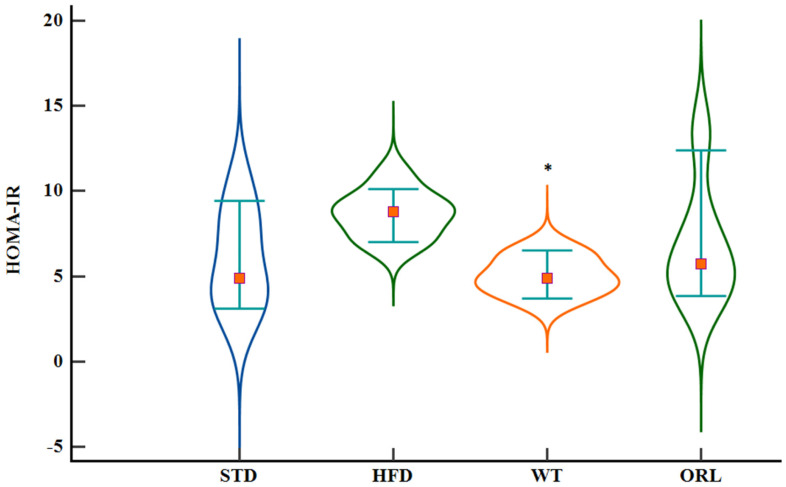
HOMA-IR values observed in the experimental cohorts following a 12-week intervention period. HOMA-IR values are shown for the standard diet (STD), high-fat diet (HFD), high-fat diet plus white tea (WT), and high-fat diet plus orlistat (ORL) groups. Data are presented as box-and-whisker plots indicating the median, interquartile range, and minimum–maximum values. * Indicates a statistically significant difference compared with the HFD group (*p* < 0.05).

A comparative analysis of insulin resistance across the groups revealed that the HFD group exhibited the highest HOMA-IR values. In contrast, the WT group presented with diminished HOMA-IR levels relative to the HFD group (*p* = 0.008) (Figure 1).

**Figure 2:**
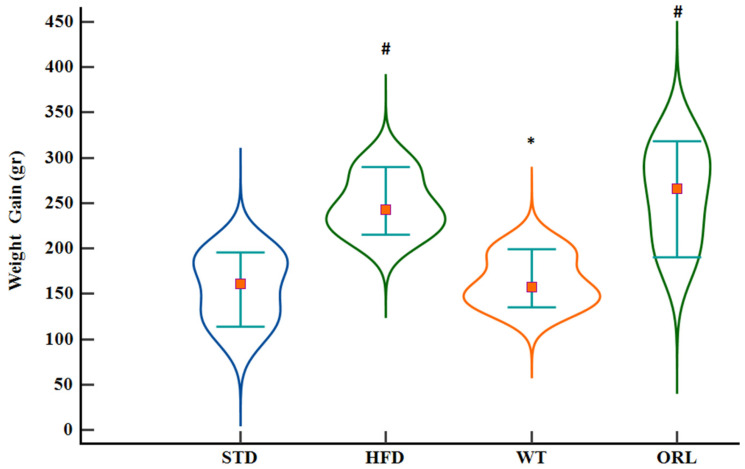
Body weight gain across experimental groups after 12 weeks of dietary intervention. Body weight gain (g) is shown for the standard diet (STD *n* = 8), high-fat diet (HFD *n* = 8), high-fat diet plus white tea (WT *n* = 8), and high-fat diet plus orlistat (ORL *n* = 8) groups. Data are presented as box-and-whisker plots indicating the median, interquartile range, and minimum–maximum values. * Indicates a statistically significant difference compared with the HFD group (after post hoc *p* = 0.044), and # indicates a statistically significant difference compared with the STD group (after post hoc *p* = 0.009).

The evaluation of body weight increase showed a greater value in the HFD and ORL groups in comparison to the control (*p* = 0.009; effect size = 0.56 and *p* = 0.012; effect size = 0.54 respectively). A minimal increase in body weight was recorded for the WT cohort, falling below that of the HFD cohort (*p* = 0.044; effect size = 0.47) (Figure 2). Detailed data regarding body weight gain and HOMA-IR levels across all experimental groups are presented in Table 1.

### 2.3. GREM1 Gene Expression in Visceral Adipose Tissue

GREM1 gene expression in visceral adipose tissue increased 1.92-fold in the high-fat diet group compared to the control group. In the WT and ORL groups, GREM1 expression levels were increased by 1.07-fold and 1.17-fold, respectively, relative to the controls. However, the differences were not found to be statistically significant (*p* = 0.063).

### 2.4. Serum and Tissue Levels of Gremlin-1 and BMP-4

**Figure 3:**
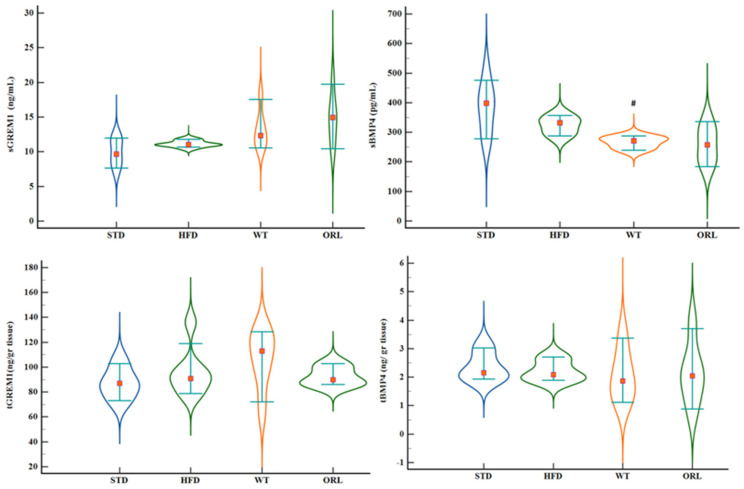
Serum and retroperitoneal adipose tissue levels of Gremlin-1 and BMP-4 across experimental groups after 12 weeks of intervention. Box-and-whisker plots depict serum Gremlin-1 (sGREM1), serum BMP-4 (sBMP4), tissue Gremlin-1 (tGREM1), and tissue BMP-4 (tBMP4) in the standard diet (STD), high-fat diet (HFD), high-fat diet plus white tea (WT), and high-fat diet plus orlistat (ORL) groups. Data are presented as median, interquartile range, and minimum–maximum values. # Indicates a statistically significant difference compared with the STD group (*p* < 0.05).

Serum and tissue levels of Gremlin-1 and BMP-4 were evaluated across experimental groups. The WT group demonstrated increased serum and tissue Gremlin-1 levels; however, these variations lacked statistical significance. Comparisons of serum Gremlin-1, tissue Gremlin-1, and tissue BMP-4 levels between groups yielded similar values (*p* = 0.043, *p* = 0.282, and *p* = 0.791, respectively). The initial disparity in serum Gremlin-1 levels did not attain statistical significance after the post hoc analysis. In contrast, serum BMP-4 levels in the WT group were lower than those observed in the STD group (*p* = 0.012; effect size = 0.48) (Figure 3), however, no statistically significant disparities were observed in tissue BMP-4 concentrations across the various cohorts.

### 2.5. Correlation Analysis

Correlation analyses revealed a moderate, negative, and statistically significant association between the serum Gremlin-1 and serum BMP-4 levels (Spearman’s rho = −0.493, *p* = 0.011). In addition, the serum BMP-4 levels showed a moderate correlation with HOMA-IR values (Spearman’s rho = 0.564, *p* = 0.003). No other significant correlations were identified among the analyzed parameters.

## 3. Discussion

The present investigation elucidates exploratory findings regarding the prophylactic impact of white tea supplementation in the context of early diet-induced metabolic dysregulation. In this non-obese high-fat diet model, white tea administration was associated with lower weight gain and lower HOMA-IR values compared to high-fat feeding alone. These differences coincided with alterations in circulating components of the Gremlin-1/BMP-4 axis; however, given the lower food intake observed in the WT group, these findings should be interpreted as intake-associated observations rather than evidence of direct metabolic pathway modulation. This observation is consistent with an association between nutrient intake and early metabolic markers. Further research is needed to confirm gene-expression and signaling control.

### 3.1. Metabolic Effects of White Tea Supplementation and Consistency with the Literature

The attenuated weight gain observed in the WT group compared with the HFD group is in line with previous studies reporting associations between tea consumption and alterations in energy metabolism [32,33,34]. Existing literature suggests that tea-derived polyphenols may influence energy expenditure, lipid utilization, and adipogenic processes [35,36,37,38]. In parallel, the lower HOMA-IR values in the WT group relative to the HFD group indicate an improvement in insulin sensitivity that coincided with these metabolic changes. This finding is consistent with previous reports indicating that white tea polyphenols, including epigallocatechin gallate (EGCG), have been associated with improvements in insulin signaling in experimental models [39,40,41,42]. EGCG may influence glucose metabolism through the activation of AMP-activated protein kinase (AMPK). EGCG might also change inflammation in fat tissue. Both of these actions affect how insulin works. Although individual bioactive compounds were not directly assessed in the present study, the observed reduction in HOMA-IR is biologically plausible in light of these previously reported mechanisms and supports consistency between our findings and the existing literature. Although these findings are consistent with previous studies, an important consideration should not be overlooked. In the present study, all animals were fed ad libitum, and no intentional caloric restriction or feeding intervention was applied. Despite this design, animals in the WT group were observed to consume less food than those in the other experimental groups. However, as food intake was not defined as a primary outcome measure at the study design stage, this observation could not be systematically quantified or subjected to formal statistical analysis. Nevertheless, this pattern raises the possibility that white tea may influence feeding behavior or appetite regulation. Given the scope and design of the current study, this observation remains exploratory and hypothesis-generating, and its investigation was beyond the objectives of the present work, warranting dedicated evaluation in future studies. The lack of reduced body weight gain in the orlistat (ORL) group represents an important consideration in the interpretation of our findings. Despite its established pharmacological action on fat absorption, orlistat did not lead to lower weight gain under ad libitum high-fat feeding conditions in the present non-obese model. This observation highlights that pharmacological inhibition of dietary fat absorption does not necessarily translate into reduced weight gain in all experimental settings, particularly when caloric intake remains high. Accordingly, the behavior of the ORL group is considered a potential confounder and a limitation of the study.

### 3.2. The Gremlin-1/BMP-4 Axis: Concordant Correlation Findings

The inverse relationship observed between Gremlin-1 and BMP-4 in serum aligns with Gremlin-1′s known function as a standard BMP inhibitor [43,44,45,46]. Gremlin-1 is known to bind BMP-4 at the ligand level, thereby limiting signal transmission, and thus an inverse relationship between their circulating levels represents an expected finding [47,48]. In addition, the positive correlation between serum BMP-4 and HOMA-IR suggests that BMP-4 may behave as a marker associated with metabolic stress and insulin resistance; however, this interpretation should be considered a preliminary inference at this stage. Previous studies found higher BMP-4 levels in people with obesity. This was also seen in those with metabolic syndrome and type 2 diabetes. These higher levels matched insulin resistance. From this perspective, our correlation analyses are supportive of existing knowledge.

### 3.3. Interpretation of Findings Partially Inconsistent with or Unexpected in the Literature

In the present study, although GREM1 gene expression in visceral adipose tissue showed a 1.92-fold increase in the HFD group, this increase did not reach statistical significance (*p* = 0.063), appearing partially inconsistent with literature reports that typically describe an upregulation of Gremlin-1 in response to HFD exposure. Nevertheless, this finding may be explained by several factors. First, our study represents an early-stage, pre-obesity model, in which metabolic stress may not yet have progressed to a sufficiently chronic state. In addition, the sample size, particularly in the context of borderline statistical significance, may have limited the power to detect a definitive difference. The heterogeneous nature of visceral adipose tissue and the presence of early compensatory mechanisms may also account for the relatively modest increase in Gremlin-1 expression.

Likewise, the numerically higher serum and tissue Gremlin-1 levels observed in the WT group, despite not reaching statistical significance, do not fully align with the expected pattern described in the literature. While a reduction in Gremlin-1 levels might be anticipated in the context of metabolic improvement, this observation may be interpreted through several possible mechanisms. The higher Gremlin-1 levels observed in the WT group may reflect context-dependent regulatory variability rather than a direct effect of white tea. Alternatively, circulating Gremlin-1 concentrations may not directly mirror tissue-level biological activity. Considering the lower food intake in the WT group and the exploratory design, these observations should be regarded as hypothesis-generating rather than mechanistic evidence. Serum BMP-4 levels were found to be lower in the WT group compared to the STD group. This finding seems contradictory. BMP-4 is usually considered metabolically protective. Lower levels of this factor in a metabolically improved group are unexpected. However, the similarity of tissue BMP-4 levels across groups suggests the existence of distinct regulatory mechanisms operating at the systemic and tissue levels [48,49,50,51,52,53,54]. This observation may reflect compartment-specific regulation and/or early adaptive responses; confirmation would require receptor/downstream readouts (e.g., SMAD activation), which were not assessed here [47,54].

### 3.4. Refined Conceptual Framework and Hypothesis

When considered collectively, our findings indicate that white tea supplementation is associated with improved insulin sensitivity and attenuated weight gain in an early-stage high-fat diet-induced metabolic dysregulation model. These metabolic improvements appear to coincide with selective changes in circulating Gremlin-1/BMP-4-related markers, interpreted as exploratory associations rather than evidence of definitive pathway modulation, given the absence of consistent tissue-level effects and downstream signaling analyses. Rather than indicating classical Gremlin-1/BMP-4 antagonistic signaling, the observed patterns may involve changes in the efficiency of BMP signaling and/or contributions from BMP-independent pathways that have been implicated in metabolic regulation in previous studies, including AMPK-related processes, PPAR-γ-associated modulation, adipokine signaling, and inflammatory regulation. This conceptual framework offers a plausible explanation for the persistence of metabolic improvement despite reduced circulating BMP-4 levels, while emphasizing the exploratory and hypothesis-generating nature of these interpretations.

### 3.5. Limitations

Several limitations of the present study should be acknowledged. Firstly, markers of beige or brown adipocyte differentiation were not assessed, precluding a direct evaluation of adipose tissue browning. Secondly, despite the observed changes in the circulating components of the Gremlin-1/BMP-4 axis, receptor-level signaling and downstream SMAD activation were not examined. Furthermore, measurements pertaining to alternative signaling pathways, including AMPK, PPAR-γ, and adiponectin, were not included. Thirdly, the sample size was small. This may have limited our ability to find small changes. These changes were in gene and protein expression in fat tissue. Fourthly, the exploratory nature of the study and the absence of a formal power analysis may limit the ability to detect subtle biological effects. Accordingly, the observed associations should be interpreted cautiously and viewed as preliminary signals requiring confirmation in larger, adequately powered investigations. Finally, the timing of orlistat and white tea administration was not synchronized with feeding rhythms, which may have influenced comparative efficacy. Despite these limitations, the present study offers insight into the preventive metabolic actions of white tea. By combining metabolic phenotyping with circulating and tissue-level gene expression-related markers, our findings suggest exploratory associations between white tea intake and early diet-induced metabolic changes, rather than definitive evidence of signaling pathway modulation. The observed interplay between Gremlin-1 and BMP-4 highlights a novel molecular framework through which white tea may exert insulin-sensitizing and weight-attenuating effects. Although orlistat was included as a pharmacological comparator due to its established use in obesity management, it does not directly target the Gremlin-1/BMP-4 signaling axis. Mechanistic interpretation is limited. This is because there is no pathway-specific modulator. Future studies could offer more definitive insights. These studies should use selective Gremlin-1 or BMP-4 interventions.

## 4. Materials and Methods

### 4.1. Experimental Animals and Study Design

The experimental methodology utilized 32 male Sprague–Dawley rats, with an age range of 6–8 weeks and an initial body weight of 150–200 g. All experimental procedures were approved by the Recep Tayyip Erdoğan University Animal Experiments Local Ethics Committee in 2020 (Decision No: 2020/29). All experimental procedures, animal handling, group allocation, outcome measures, and statistical analyses were performed and reported in accordance with the ARRIVE (Animal Research: Reporting of In Vivo Experiments) guidelines and key checklist items were considered during manuscript preparation.

Animals were kept in a pathogen-free facility. The room temperature was 22 ± 2 °C. The humidity was 55–60%. A light and dark cycle was maintained for 12 h each. All animals were allowed ad libitum access to food and water throughout the study period. After a week of acclimatization on a standard laboratory chow, rats were randomly divided into four experimental groups (*n* = 8 per group). The study encompassed four groups: a control group receiving a standard laboratory diet (STD), a high-fat diet group (HFD), a high-fat diet group administered 30 mg/kg/day of orlistat (ORL), and a high-fat diet group supplemented with 5 mg/kg/day of white tea (WT). Except for the control group, animals received white tea for 12 weeks, after which metabolic parameters were assessed. The high-fat diet was formulated to provide 45% of the total caloric intake from fat (Arden Research & Experimental Co., Sincan, Ankara, Türkiye). The composition of the diets is detailed in Table 2. The control group received a standard pellet diet (Bayramoğlu Yem, Erzurum, Türkiye). Body weight was recorded weekly throughout the experimental period. All interventions were administered once daily by oral gavage at the same time each morning. At the end of the experimental period, animals were fasted for 12 h prior to sacrifice.

Animals were randomly allocated to experimental groups using a simple randomization procedure prior to the start of dietary intervention. Group allocation was concealed from investigators performing biochemical analyses and data evaluation. Due to the nature of dietary administration and daily gavage procedures, blinding during intervention was not feasible; however, outcome measurements were performed using coded samples. No predefined exclusion criteria were applied, and all animals that completed the study were included in the final analysis. Sample size was determined based on prior similar experimental studies and practical considerations, and the study was designed as an exploratory investigation rather than a formally powered efficacy trial.

### 4.2. Preparation and Administration of White Tea and Orlistat

White tea samples were obtained from the General Directorate of Tea Enterprises (ÇAYKUR, Rize, Türkiye). Tea leaves were harvested exclusively during the first flush in May, in accordance with standard manufacturing practices. White tea infusion was prepared fresh daily by brewing 25 mg of dried white tea leaves in 250 mL of distilled water at a constant temperature of 90 °C for 10 min in a glass container. Following infusion, the solution was filtered and rapidly cooled to room temperature by placing the glass container in an ice bath. The freshly prepared and cooled infusion was administered immediately by oral gavage at a dose of 5 mg/kg/day (tea leaf equivalent), based on previously published experimental studies.

Orlistat was administered orally by gavage at a dose of 30 mg/kg/day. Control and high-fat diet groups received an equivalent volume of distilled water (1 mL/day) by oral gavage to ensure the consistency of handling across groups.

### 4.3. Sample Collection

In this study, retroperitoneal adipose tissue was analyzed as a representative visceral adipose tissue depot. At the end of the study, animals were anesthetized by intraperitoneal injection of ketamine hydrochloride (50 mg/kg; Ketalar^®^, Pfizer, İstanbul, Türkiye) and xylazine hydrochloride (10 mg/kg; Rompun^®^, Bayer, Germany). Blood samples were collected via intracardiac puncture into serum separator tubes and allowed to clot at room temperature. Samples were then centrifuged at 1500× *g* for 15 min at 4 °C, and serum aliquots were stored at −80 °C until analysis. Retroperitoneal adipose tissue samples were excised immediately after sacrifice, rinsed with cold saline, snap-frozen in liquid nitrogen, and stored at −80 °C for subsequent molecular and biochemical analyses.

### 4.4. Preparation of Serum and Tissue Homogenates

Serum samples were thawed only once prior to biochemical analyses. For tissue homogenization, retroperitoneal adipose tissue samples were processed using a homogenization buffer containing 20 mM sodium phosphate and 140 mM potassium chloride (pH 7.4). Tissue samples (0.1 g) were homogenized in 1 mL of buffer and centrifuged at 800× *g* for 10 min at 4 °C. Supernatants were collected and stored at −80 °C until analysis.

### 4.5. Biochemical Analyses

Fasting plasma glucose levels were measured using an enzymatic hexokinase method on a Beckman Coulter AU6000 autoanalyzer (Beckman Coulter, Indianapolis, IN, USA) and expressed in mg/dL. The analytical coefficient of variation (CV) for glucose measurements was <3%. Serum insulin concentrations were determined using a commercially available ELISA kit (SinoGeneClon Biotech Co., Ltd., Hangzhou, China) according to the manufacturer’s instructions. The analytical performance characteristics of the insulin assay included a within-run CV of <8%, a between-run CV of <10%, and an analytical sensitivity of 0.2 mU/L. Insulin resistance was assessed using the Homeostatic Model Assessment for Insulin Resistance (HOMA-IR) index, calculated using the formula: HOMA-IR = (fasting glucose × fasting insulin)/405. Serum levels of Gremlin-1 (Cat. No: SG-21730), BMP-4 (Cat. No: SG-20681), and tissue levels of Gremlin-1 and BMP-4 were determined using the same ELISA methodology.

### 4.6. RNA Isolation and Quantitative Real-Time PCR

Total RNA was extracted from retroperitoneal adipose tissue using the High Pure RNA Isolation Kit (Roche Diagnostics, Mannheim, Germany) according to the manufacturer’s protocol. RNA concentration and purity were assessed using a Thermo Multiskan Go spectrophotometer with µDrop plates (Thermo Fisher Scientific, Waltham, MA, USA). Samples with A260/A280 ratios between 1.8–2.1 were considered acceptable for downstream analysis. Complementary DNA synthesis was executed utilizing 1000 ng of total RNA within a 20 µL reaction volume, employing the High-Capacity cDNA Reverse Transcription Kit (Applied Biosystems, Waltham, MA, USA). Quantitative real-time PCR (qRT-PCR) was conducted using a LightCycler 480 II system (Roche, Basel, Switzerland) with LightCycler 480 Probes Master mix. Each reaction contained 30 ng of cDNA in a total volume of 20 µL. Gene-specific probes for GREM1 and GAPDH (Thermo Fisher Scientific, Waltham, MA, USA) were used. GAPDH served as the reference gene for normalization, and relative gene expression was calculated using the ΔΔCt method.

### 4.7. Characterization of White Tea Composition

The phenolic composition of white tea was previously characterized using high-performance liquid chromatography with diode-array detection (HPLC-DAD). Quantitative results are presented in Table 3. Epigallocatechin (EGC) and epigallocatechin gallate (EGCG) were identified as the predominant catechin derivatives in the white tea samples.

### 4.8. Data Processing and Analytical Pipeline

An a priori analytical pipeline was implemented to ensure standardized, reproducible, and integrated data processing across metabolic and molecular outcomes. After randomization and intervention, animal-level phenotypic data were quantified under standardized fasting conditions, including body weight gain, cumulative food intake, and insulin resistance (HOMA-IR). Serum and tissue biomarkers (Gremlin-1 and BMP-4) were quantified via enzyme-linked immunosorbent assays under standardized pre-analytical and analytical parameters, and visceral adipose tissue gene expression was evaluated via a predetermined qRT-PCR protocol. The datasets were subjected to quality control, distribution-based statistical analysis with correction for multiple comparisons, and correlation analyses to integrate metabolic phenotypes with gene expression signaling parameters.

### 4.9. Statistical Analysis

Statistical analyses were performed using IBM SPSS Statistics (version 29.0; SPSS Inc., Chicago, IL, USA) and MedCalc^®^ Statistical Software version 23.4.4 (MedCalc Software Ltd., Ostend, Belgium; https://www.medcalc.org; 2025). Data distribution was assessed using non-parametric methods. For continuous variables, the data were displayed as the median value, bracketed by the minimum and maximum observations. Comparisons between groups were performed using the Kruskal–Wallis test followed by post hoc Dunn test with Bonferroni correction for multiple comparisons. Correlations between variables were evaluated using Spearman’s rank correlation analysis. Differences were considered statistically significant at *p* < 0.05.

## 5. Conclusions

In conclusion, white tea supplementation was associated with attenuated body weight gain and preserved insulin sensitivity in a non-obese high-fat diet model, as reflected by reduced weight gain and lower HOMA-IR values compared with high-fat feeding alone. These metabolic differences coincided with selective changes in circulating Gremlin-1/BMP-4-related markers and should be interpreted as exploratory biomarker-level associations rather than evidence of signaling pathway modulation. Although tissue-level alterations were modest and mechanistic pathways could not be directly delineated within the scope of this study, the observed systemic patterns support the biological plausibility of white tea influencing early diet-induced metabolic responses. These findings suggest that white tea intake may be associated with early metabolic adaptations in this exploratory model at the level of circulating biomarkers rather than confirmed signaling pathway regulation. Further studies incorporating pathway-specific signaling analyses and translational approaches are warranted to validate these observations and clarify their relevance to human metabolic health.

## Figures and Tables

**Table 1:** Metabolic, biochemical, and molecular parameters across experimental groups after 12 weeks of intervention. The table summarizes the serum and tissue levels of Gremlin-1 (sGREM1, tGREM1), serum and tissue BMP-4 (sBMP4, tBMP4), insulin resistance estimated by HOMA-IR, and body weight gain in the standard diet (STD), high-fat diet (HFD), high-fat diet plus white tea (WT), and high-fat diet plus orlistat (ORL) groups. We show the data as the median (min–max). Overall group comparisons were performed using the Kruskal–Wallis test.

	STD	HFD	WT	ORL	*p* ‡
sGREM1(ng/mL)	9.64 (7.19–12.14)	11 (10.45–11.93)	12.35 (10.36–18.3)	14.94 (10.1–20.57)	0.043
tGREM1(ng/grtissue)	87.15 (70.95–107.15)	90.8 (76.8–136.15)	113.025 (66.3–129.25)	89.825 (85.9–103.6)	0.282
sBMP4(pg/mL)	398 (277–513)	332 (278–365)	271 (237–289) #	257 (177–344)	0.012
tBMP4(ng/grtissue)	2.15 (1.91–3.18)	2.09 (1.87–2.74)	1.865 (1.05–3.55)	2.04 (0.74–3.95)	0.791
HOMA-IR	4.9 (2.29–10.57)	8.76 (7.01–10.95)	4.9 (3.56–6.61) *	5.715 (3.72–13.42)	0.040
Weight gain (gr)	161 (102–199)	243 (208–295) #	157.5 (134–200) *	266 (185–321) #	0.001

Data were given as median (min–max) ‡: Kruskal–Wallis test; * WT versus HFD group after post hoc analysis (for HOMA-IR *p* = 0.045, for weight gain *p* = 0.044), and # compared with the STD group after post hoc analysis (for sBMP-4 *p* = 0.041, for weight gain HFD *p* = 0.009, ORL *p* = 0.012).

**Table 2:** Composition and nutritional characteristics of the experimental diets. The table presents the detailed ingredient composition and nutritional content of the standard control diet and the high-fat diet used in the study. For the control diet, macro- and micronutrient contents are reported as percentages or concentrations per kilogram. For the high-fat diet, individual ingredients are listed with their respective amounts (g/kg) and caloric contributions, resulting in a total energy density of 4500 kcal/kg with 22% fat and 20% protein.

Company	Name/Code	Content	
		Ingredient	Amount/Calories
Bayramoğlu Yem ve Un Sanayi Tic. A.Ş, İstanbul, Türkiye	Control feed	Humidity	12.8%
		Raw protein	23%
		Raw fat	1.7%
		Raw cellulose	3.7%
		Raw ash	8.3%
		Sodium	0.5%
		Vitamin A	12,000,000 IU/kg
		Manganese (manganese sulfate)	95 mg/kg
		Iron (iron sulfate monohydrate)	31 mg/kg
		Zinc (zinc oxide)	95 mg/kg
		Cobalt (cobalt carbonate)	0.5 mg/kg
		Selenium (sodium selenite)	0.3 mg/kg
		Iodine (calcium iodate anhydrous)	2.28 mg/kg
Arden Research & Experimental Co., Sincan, Ankara, Türkiye	High-fat diet feed (22% fat, 20% protein, total 4500 kcal)	Casein	200 g/kg (800 kcal)
		Corn starch	109 g/kg (410.9 kcal)
		Dextrinized starch	193 g/kg (780 kcal)
		Sugar	121.5 g/kg (486 kcal)
		Palm oil	220 g/kg (1980 kcal)
		Cellulose	50 g/kg
		Mineral mixture (S10026)	10 g/kg
		Vitamin mixture (V10001)	10 g/kg (40 kcal)
		L-cystine	3 g/kg
		Choline bitartrate	2.5 g/kg (12 kcal)
		DCP (dicalcium phosphate)	13 g/kg
		Calcium carbonate	5.5 g/kg
		Potassium citrate monohydrate	16.5 g/kg
		Methyl paraben	0.014 g/kg
		Aromatic chemicals	48 g/kg

**Table 3:** Phenolic and alkaloid composition of white tea determined by HPLC-DAD analysis. The table shows the major constituents identified in white tea, including catechins, caffeine, and gallic acid, with their corresponding retention times and concentrations. Quantitative data are presented as mean ± standard deviation based on duplicate measurements (*n* = 2). Compounds not detected under the applied analytical conditions are indicated by a dash (–).

Number	Compound Name	Retention Time (Min)	Concentration (*n* = 2, µg/g)
1	Gallic acid	3.202	5.55 ± 0.61
2	Epigallocatechin	4.499	261.4 ± 3.59
3	Catechin	5.488	–
4	Caffeine	7.750	101.51 ± 2.61
5	Epigallocatechin 3-gallate	8.678	119.8 ± 2.31
6	Epicatechin	9.800	–
7	Epicatechin 3-gallate	15.916	46.36 ± 1.70

## Data Availability

The datasets used are available from the corresponding author on reasonable request.
